# Using genetics to understand the role of kidney function in COVID-19: a mendelian randomization study

**DOI:** 10.1186/s12882-021-02586-6

**Published:** 2021-11-13

**Authors:** Jie V. Zhao, C. Mary Schooling

**Affiliations:** 1 School of Public Health, Li Ka Shing Faculty of Medicine, The University of Hong Kong, 1/F, Patrick Manson Building, 7 Sassoon Road, Hong Kong, SAR China; 2 City University of New York, School of Public Health and Health policy, New York, NY USA

## Abstract

**Background:**

Kidney dysfunction occurs in severe COVID-19, and is a predictor of COVID-19 mortality. Whether kidney dysfunction causes severe COVID-19, and hence is a target of intervention, or whether it is a symptom, is unclear because conventional observational studies are open to confounding. To obtain unconfounded estimates, we used Mendelian randomization to examine the role of kidney function in severe COVID-19.

**Methods:**

We used genome-wide significant, uncorrelated genetic variants to predict kidney function, in terms of estimated glomerular filtration rate (eGFR) and urine albumin-to-creatinine ratio (UACR), and then assessed whether people with genetically instrumented higher eGFR or lower UACR, an indication of better kidney function, had a lower risk of severe COVID-19 (8779 cases, 1,001,875 controls), using the largest available cohorts with extensive genotyping. For comprehensiveness, we also examined their role in COVID-19 hospitalization (24,274 cases, 2,061,529 controls) and all COVID-19 (1,12,612 cases, 2,474,079 controls).

**Results:**

Genetically instrumented higher eGFR was associated with lower risk of severe COVID-19 (odds ratio (OR) 0.90, 95% confidence interval (CI) 0.83, 0.98) but not related to COVID-19 hospitalization or infection. Genetically instrumented UACR was not related to COVID-19.

**Conclusions:**

Kidney function appears to be one of the key targets for severe COVID-19 treatment. Use of available medications to improve kidney function, such as antihypertensives, might be beneficial for COVID-19 treatment, with relevance to drug repositioning.

**Supplementary Information:**

The online version contains supplementary material available at 10.1186/s12882-021-02586-6.

## Background

Pandemic COVID-19 can cause multi-organ dysfunction [1], which imposes unprecedented pressure on the healthcare system. Mortality from COVID-19 is a major concern in global health. Identifying factors affecting severe COVID-19 provides insight for identifying potential targets for COVID-19 treatment. Kidney function might be one of the factors involved in severe COVID-19. Kidney function may induce systematic inflammation and immunosuppression [2]. A cytokine storm and immunosuppression are key features of severe COVID-19 [3]. Kidney dysfunction is one of the typical characteristics of severe COVID-19 [4, 5]. A large study in 17 million patients, covering 40% of all patients in England, suggested that kidney dysfunction, indicated by lower eGFR, was associated with higher COVID-19 mortality [6]. Similarly, a study in China also found kidney dysfunction, indicated by higher serum creatinine, was associated with COVID-19 in-hospital deaths [7]. Understanding the role of kidney function in COVID-19 would be of great value for identifying new treatment strategies for COVID-19. However, whether kidney function affects COVID-19 severity, or is instead a symptom rather than a target of intervention, has not been examined [8]. Conventional observational studies cannot provide a definitive answer due to unavoidable confounding by factors such as socioeconomic position and use of medications.

In this situation, Mendelian randomization (MR) provides a way to obtain unconfounded estimates without any intervention. MR utilizes genetic variants as instrument to predict the exposure [9]. As the genetic predictors are determined at conception, they are less likely to be affected by socioeconomic position or use of medication, thereby minimizing confounding [9]. To clarify the role of kidney function in COVID-19, in this MR study we examined whether people with genetically instrumented better kidney function, specifically genetically instrumented higher estimated glomerular filtration rate (eGFR) or lower urine albumin-to-creatinine ratio (UACR), had lower risk of severe COVID-19. To check for reverse causality, we assessed the role of severe COVID-19 in kidney function using MR. For comprehensiveness, we also considered the role of kidney function in COVID-19 hospitalization and infection.

## Methods

We used a two-sample MR study to obtain unconfounded associations. Specifically, we used published, genome-wide significant (*p*-value< 5 × 10^− 8^), uncorrelated (r^2^ < 0.05) genetic variants to predict eGFR and UACR, and then obtained their associations with severe COVID-19 (8779 cases, 1,001,875 controls) based on several large studies such as the UK Biobank, GENCOVID, genomiCC, and BRACOVID [10]. We also examined their associations with COVID-19 hospitalization (24,274 cases, 2,061,529 controls) and all COVID-19 (1,12,612 cases, 2,474,079 controls) using the largest publicly available COVID-19 genome wide association study (GWAS), largely of people of European ancestry (details of the participating studies shown in https://www.covid19hg.org/results/r6/*).*

### Genetic instruments for eGFR and UACR

Genetic instruments for eGFR and UACR were extracted from the latest GWAS [11, 12]. Specifically, genetic predictors for eGFR were published genetic variants in the trans-ethnic GWAS of eGFR (standardized and log transformed) provided by the CKDGen Consortium, conducted in 765,348 people, 567,460 of European ancestry, 50% men, with median age 54 years and median eGFR 89 mL min^− 1^ per 1.73 m^2^ (interquartile range (IQR): 81, 94) [11]. To avoid population stratification, we only used genetic variants reaching genome-wide significance in people of European ancestry. The GWAS controlled for age, sex, genetic principal components, relatedness and other study-specific characteristics as appropriate [11]. We selected independent (r^2^ < 0.05) genome wide significant genetic predictors using the “ld_clump” function of MR-base. The genetic predictors for UACR were extracted from the most recent GWAS of UACR in the UK Biobank, in 437,027 people with UACR measured and of European ancestry, with replication in the EXTEND study (*n* = 5679) [12]. UACR was calculated from urinary albumin and creatinine, and inverse-normalized. Urinary albumin lower than the assay detection limit (6.7 mg/L in the UK Biobank) was set at 6.7 mg/L [12]. The GWAS used a linear-mixed model, adjusted for age, sex, study centre and genotyping array [12].

### Genetic associations with COVID-19

The summary statistics in the GWAS were provided by the COVID-19 host genetics initiative round 6 (https://www.covid19hg.org/results/), based on large cohort studies [10]. The majority of studies were conducted in Europe (55%) and the US (28%), amongst which the United Kingdom (10%) and Italy (9%) are the largest [10]. Genetic associations with severe COVID-19, COVID-19 hospitalization and all COVID-19 were obtained from GWAS using the following case definitions. Severe COVID-19 was defined as death or respiratory support following hospitalization with COVID-19 as the primary reason for admission. COVID-19 hospitalization was defined as hospitalization due to corona-related symptoms, with laboratory confirmed SARS-CoV-2 infection. All COVID-19 was defined as 1) laboratory confirmed SARS-CoV-2 infection (RNA and/or serology based), or 2) physician diagnosis of COVID-19, or 3) self-report as COVID-19 positive. The controls were participants in these cohorts who are not cases. The GWAS was adjusted for age, age square, sex, the interaction of age and sex and principal components [13].

### The role of severe COVID-19 in kidney function

To assess the role of severe COVID-19 in kidney function, we used independent (r^2^ < 0.05) genetic variants related to severe COVID-19 at genome wide significance as instruments [14] applied to GWAS of eGFR and UACR. Six genetic variants at genome-wide significance were identified from a GWAS meta-analysis including critically ill patients of European descent from Genetics Of Mortality In Critical Care (1676 cases, 8380 controls), COVID-19 Host Genetics Initiative (2415 cases, 477,741 controls) and 23andMe (1128 cases, 679,531 controls) [14]. Genetic associations of these genetic variants with kidney function were obtained from GWAS of eGFR and UACR as given above [11, 12].

### Statistical analysis

MR estimates were based on the Wald estimates [15], i.e., genetic association with the outcome (primarily severe COVID-19, and secondarily COVID-19 hospitalization or infection) divided by the genetic association with eGFR or UACR. The genetic variant specific Wald estimates were meta-analyzed using inverse variance weighting (IVW), with multiplicative random effects in univariable MR. As eGFR may affect survival [16], and COVID-19 is affected by prior comorbidities and some common risk factors (such as smoking and socioeconomic position) [6, 17], the MR study on eGFR and COVID-19 might be open to selection bias [18]. To control for such bias, when assessing the role of eGFR in COVID-19 we controlled for smoking initiation and education using multivariable MR [18]. We did not do this for UACR because UACR does not affect mortality [19]. BMI also plays a role in COVID-19, so the eGFR genetic predictors could affect BMI and thereby COVID-19 independent of eGFR, i.e., be a confounder of eGFR on COVID-19, alternatively BMI could be a downstream consequence of eGFR. To address these possibilities, we additionally controlled for BMI in multivariable MR. In the multivariable MR, we additionally included genetic variants predicting smoking [20] and education (proxied by years of schooling) [21], and removed duplicate and correlated (r^2^ > 0.05) genetic variants among those predicting kidney function, smoking or education. In sensitivity analysis, we additionally included genetic predictors for BMI, to additionally control for BMI. We obtained genetic associations with smoking initiation from the UK Biobank summary statistics (http://www.nealelab.is/uk-biobank) adjusted for age, sex, age^2^, interaction of sex with age, and age^2^, and the first 20 principal components, and the genetic associations with education from the relevant GWAS in of 766,345 people of European ancestry controlling for age, sex, interaction of age and sex, and 20 principal components [21].

In sensitivity analysis, we used different methods with different assumptions, considering the potential bias from pleiotropic genetic effects (i.e. a genetic predictor being related to COVID-19 other than via kidney function [22]). Specifically, we used a weighted median (in univariable and multivariable MR) and MR-Egger (in univariable and multivariable MR). A weighted median can provide consistent estimates even when up to 50% of the information comes from invalid genetic variants [23]. MR-Egger detects potential pleiotropy from the significance of its intercept [24], which are less vulnerable to pleiotropy.

Similarly, we used IVW with multiplicative random effects to assess the role of severe COVID-19 in kidney function in the main analysis, and conducted sensitivity analyses using a weighted median and MR-Egger. All statistical analyses were conducted using R version 4.0.1 (R Foundation for Statistical Computing, Vienna, Austria), and the R package “MendelianRandomization”. This analysis of publicly available data does not require ethical approval.

## Results

We identified 230 uncorrelated genetic variants predicting eGFR in people of European ancestry. When assessing the role of eGFR in severe COVID-19, we additional included 350 genetic predictors for smoking initiation and 613 genetic predictors for education whose associations with severe COVID-19, eGFR, smoking and education were taken from the relevant GWAS. After removing duplicate and correlated genetic variants, 987 genetic variants were used in multivariable MR for eGFR and severe COVID-19. Similarly, we identified and used 995 and 996 genetic variants respectively when assessing the role of eGFR in COVID-19 hospitalization and infection. We identified 62 uncorrelated genetic variants predicting UACR; 57 of them were available in the GWAS of severe COVID-19.

**Fig. 1:**
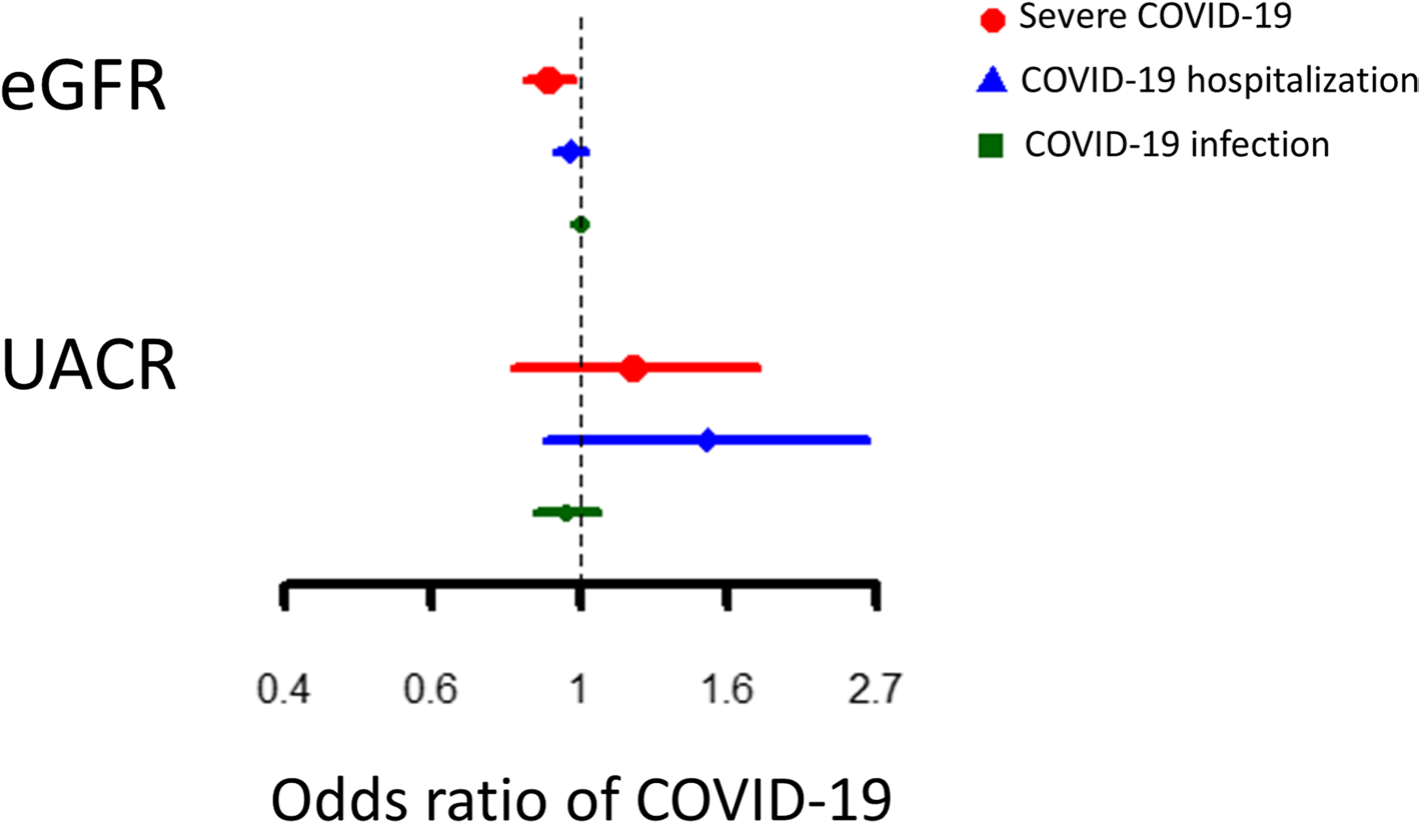
Associations of genetically instrumented kidney function with COVID-19. The estimates shown in this figure were provided in Supplemental Table. eGFR, estimated glomerular filtration rate; UACR, urine albumin-to-creatinine ratio

Genetically instrumented higher eGFR was associated with lower risk of severe COVID-19 (Fig. 1 and Supplemental Table 1). The association with COVID-19 hospitalization was in the same direction but the confidence interval included 1 (Fig. 1 and Supplemental Table 1). In sensitivity analysis, multivariable MR additionally controlling for BMI gave consistent estimates (Supplemental Table 2). Genetically instrumented higher UACR was not associated COVID-19 severity, hospitalization or infection (Fig. 1 and Supplemental Table 1); sensitivity analysis using the weighted median and MR-Egger provided similar estimates (Supplemental Table 3). Genetic instruments for severe COVID-19 were not associated with eGFR or UACR (Supplemental Table 4).

## Discussion

Our MR study for the first time shows that genetically instrumented better kidney function (based on higher eGFR), are related to lower risk of severe COVID-19, with a null association in the reverse direction. Our findings suggest that improving kidney function would be beneficial for lowering the risk of severe COVID-19, with implications for healthcare and drug repositioning.

The role of kidney function in severe COVID-19 is consistent with the sex disparity in COVID-19, where men are more vulnerable to severe COVID-19 [6], and are more vulnerable to renal failure [25]. Kidney function has multiple roles, and interacts with immune responses, inflammation, coagulation, and endothelial function [2, 26–28]. Kidney dysfunction may lead to accumulation of toxic metabolic waste and impaired protein catabolism, thereby increasing systematic inflammation and immunosuppression [2]. A cytokine storm and immunosuppression are key features of severe COVID-19 [3]. Moreover, kidney dysfunction often accompanies hypercoagulation and venous thrombosis [27, 28]. Thrombin generation, which increases the risk of thrombosis and severe COVID-19, is elevated in patients with dialysis [29]. In addition, reduced kidney function is linked to endothelial dysfunction [26], which may also lead to severe COVID-19. However, these pathways have not been clarified in experimental studies and cannot be assessed in MR studies because relevant GWAS are not available.

Despite a novel study, several limitations exist. First, these findings are preliminary and need to be interpreted cautiously. The protective association of kidney function with severe COVID-19 might be a reflection of an association specific to severe COVID-19, or a chance finding, or due to a lack of power for other COVID-19 outcomes. As the genetic predictors for kidney function only capture a small proportion of the variance [30], MR estimates are imprecise (indicated by wide confidence intervals) although less prone to confounding. As such, it would be worthwhile to replicate in a larger study. Nevertheless, these findings provide some insights for identifying new targets in COVID-19 treatment. Second, MR estimates might be confounded by population stratification, however, we restricted our analysis to genetic associations derived from people of European ancestry. Third, this study is limited to people of European descent and might not apply to other populations. However, the effects of causal factors are not expected to vary with setting, unless the relevance of the mechanism varies [31]. Fourth, MR estimates might be biased if the same samples are used to obtain genetic predictors of kidney function and their associations with COVID-19 [32]. However, the genetic predictors for eGFR were extracted from the CKDGen Consortium and their associations with COVID-19 were from several different cohorts including UK Biobank, which are not expected to overlap. More overlap for UACR is possible, given genetic predictors for UACR were derived from UK Biobank. However, all the UACR genetic predictors were genome-wide significant in the most recent GWAS [11, 12], so any bias from the overlapping would not be substantial [33]. Fifth, effects of kidney function might differ by sex, which cannot be assessed from the currently available COVID-19 GWAS summary statistics. Sixth, the definition of severe COVID-19 included death and respiratory support following hospitalization with COVID-19, the latter includes supplemental oxygen (not including simple supplementary oxygen), non-invasive mechanical ventilation and invasive mechanical ventilation. The genetic associations with severe COVID-19 were from summary statistics, and a breakdown by mode of respiratory support is not available. Replication using individual level data, where applicable, would be worthwhile. Differences in the mode of respiratory support might increase the variability of the estimation, and correspondingly widen the confidence interval.

Understanding the role of kidney function in COVID-19 is of great value for clinical practice. Kidney dysfunction leads to a higher risk of severe COVID-19, correspondingly, medications which improve kidney function might be beneficial for COVID-19, with implications for drug repositioning. Further examination of the role of medications that improve kidney function, such as ACE inhibitors, in severe COVID-19 would be worthwhile, with implications for identifying new treatment strategies for severe COVID-19.

## Conclusion

Kidney function appears to be one of the key targets for COVID-19. Exploration of the underlying pathways and use of available medications that improve kidney function, such as antihypertensives, might be beneficial for COVID-19 treatment, with relevance to drug repositioning and healthcare.

## Supplementary Information

**Additional file 1.**

## Data Availability

The dataset analysed during the current study is publicly available in https://www.covid19hg.org/results/.

## Abbreviations

CI: Confidence interval
eGFR: Estimated glomerular filtration rate
GWAS: Genome wide association study
MR: Mendelian randomization
UACR: Urine albumin-to-creatinine ratio
