# Supplementary material for: Using genetics to understand the role of kidney function in COVID-19: a mendelian randomization study
Source: BMC Nephrol. 2021 Nov 13;22:381. doi: 10.1186/s12882-021-02586-6 (PMC8590376; doi:10.1186/s12882-021-02586-6)
Supplement: Supplementary file 1 — Additional file 1. [file 12882_2021_2586_MOESM1_ESM.pdf]

Supplemental Tables

Supplemental Table 1. Associations of genetically instrumented kidney function with COVID-19

Supplemental Table 2. Associations of genetically instrumented eGFR with COVID-19 after additionally controlling for body mass index

Supplemental Table 3. Associations of genetically instrumented kidney function with COVID-19 using different analytic methods

Supplemental Table 4. Associations of genetically instrumented severe COVID-19 with kidney function

Supplemental Table 1. Associations of genetically instrumented kidney function with COVID-19

Exposure	Outcome	Method*	Controlled for	OR	95% CI	*p*	MR-Egger intercept *p*
**eGFR** (per 0.1 standard deviation of log transformed eGFR)	Severe COVID-19	MVMR-IVW	Smoking, education	0.90	0.83, 0.98	0.02	0.26
	COVID-19 hospitalization	MVMR-IVW		0.97	0.92, 1.02	0.22	0.08
	All COVID-19	MVMR-IVW		1.00	0.98, 1.02	0.64	0.32
**UACR** (per 0.1 standard deviation of inverse-normalized UACR)	Severe COVID-19	IVW	--	1.20	0.80, 1.82	0.38	0.21
	COVID-19 hospitalization	MR-Egger	--	1.54	0.89, 2.64	0.12	0.04
	All COVID-19	IVW	--	0.95	0.86, 1.06	0.37	0.40

eGFR, estimated glomerular filtration rate; UACR, urine albumin-to-creatinine ratio; IVW, inverse variance weighting.

*In the analysis on eGFR and COVID-19, we used multivariable Mendelian randomization to adjust for smoking and education to control for selection bias.

In the analysis on UACR and COVID-19 hospitalization, IVW was not used for infection because MR-Egger intercept p value is less than 0.05.

Supplemental Table 2. Associations of genetically instrumented eGFR with COVID-19 after additionally controlling for body mass index

Exposure	Outcome	Controlled for	OR	95% CI	*p*
**eGFR** (per 0.1 standard deviation of log transformed eGFR)	Severe COVID-19	Smoking, education, body mass index	0.91	0.83, 0.995	0.04
	COVID-19 hospitalization		0.98	0.93, 1.03	0.38
	COVID-19 infection		0.998	0.98, 1.02	0.84

eGFR, estimated glomerular filtration rate; OR, odds ratio; CI, confidence interval. MVMR-IVW was used in the analysis.

Supplemental Table 3. Associations of genetically instrumented kidney function with COVID-19 using different analytic methods

Exposure	Outcome	Method	OR	95% CI	*p*
**eGFR** (per 0.1 standard deviation of log transformed eGFR)	Severe COVID-19	MVMR-median	0.90	0.80, 1.01	0.08
		MVMR-Egger	0.94	0.84, 1.05	0.26
	COVID-19 hospitalization	MVMR-median	0.96	0.89, 1.03	0.26
		MVMR-Egger	1.00	0.94, 1.07	0.91
	All COVID-19	MVMR-median	1.00	0.97, 1.03	0.93
		MVMR-Egger	1.00	0.98, 1.03	0.80
**UACR** (per 0.1 standard deviation of inverse-normalized UACR)	Severe COVID-19	Weighted median	1.56	0.87, 2.78	0.14
		MR-Egger	2.02	0.81, 5.04	0.13
	COVID-19 hospitalization	Weighted median	1.09	0.79, 1.49	0.61
	All COVID-19	Weighted median	1.03	0.92, 1.16	0.56
		MR-Egger	1.03	0.84, 1.26	0.78

eGFR, estimated glomerular filtration rate; UACR, urine albumin-to-creatinine ratio; OR, odds ratio; CI, confidence interval.

Supplemental Table 4. Genetically instrumented severe COVID-19 and kidney function

Outcomes	Methods	beta	95% CI	*P* value
eGFR	IVW	0.0003	-0.001, 0.002	0.70
	Weighted median	0.0003	-0.001, 0.002	0.73
	MR-Egger	0.0001	-0.003, 0.003	0.94
UACR	IVW	-0.003	-0.01, 0.01	0.55
	Weighted median	-0.002	-0.01, 0.01	0.64
	MR-Egger	0.01	-0.01, 0.03	0.19
